# Expression of programmed cell death-ligand 1 and its correlation with clinical outcomes in gliomas

**DOI:** 10.18632/oncotarget.6884

**Published:** 2016-01-11

**Authors:** Jing Zeng, Xin-Ke Zhang, Hua-Dong Chen, Zhi-Hai Zhong, Qiu-Liang Wu, Su-Xia Lin

**Affiliations:** ^1^ State Key Laboratory of Oncology in South China, Sun Yat-sen University Cancer Center, Guangzhou, China; ^2^ Department of Pathology, Sun Yat-sen University Cancer Center, Guangzhou, China; ^3^ Department of Pediatric Surgery, The First Affiliated Hospital, Sun Yat-sen University, Guangzhou, China

**Keywords:** PD-L1, prognosis, DFS, OS, gliomas

## Abstract

Programmed cell death-ligand 1(PD-L1) was expressed in various malignancies, and interaction with its receptor programmed cell death 1 (PD-1) often contributed to immune evasion of tumor cells. In this study, we explored the expression of PD-L1 and its correlation with clinical outcomes in gliomas.

Clinicopathological data of 229 patients with gliomas was collected. PD-L1 expression was assessed by tissue-microarray-based immunohistochemistry. Over 5% of tumor cells with cytoplasm or membrane staining was defined as PD-L1 positive expression. The associations of clinicopathological features with overall survival (OS) and disease-free survival (DFS) were analyzed by univariate analysis and multivariate analysis was further performed by Cox regression model.

PD-L1 positive expression was observed in 51.1% gliomas patients and no significant association was verified between PD-L1 expression and pathological grade in 229 gliomas patients. However, PD-L1 expression rate was 49.2%, 53.7% and 68.8% for grade II, III and IV in 161 patients with those ≥ 12 months of OS, respectively. Although no significant discrepancies was displayed, there was a certain degree of differences between PD-L1 expression and pathological grade (49.2% vs. 53.7% vs. 68.8%, P = 0.327). Univariate analysis showed that PD-L1 expression was significantly associated with poor OS in the patients with long-time survival or follow up (OS ≥ 12 months) (P = 0.018), especially in patients with grade IV (P = 0.019). Multivariate analysis revealed that a strong tendency towards statistical significance was found between PD-L1 expression and poor OS (P = 0.081).

In gliomas patients with long-time survival or follow up, PD-L1 positive expression could indicate the poor prognosis and it is possible that immunotherapy targeting PD-L1 pathway needed to be determined in the further study.

## INTRODUCTION

Gliomas are the most common brain tumor and they were categorized into low-grade gliomas [pilocytic astrocytoma (grade I), diffuse astrocytoma (grade II)], and high-grade gliomas [anaplastic astrocytoma (grade III), and glioblastoma multiform (grade IV)] according to the following criteria including cell density, cell atypia, mitoses and presence or absence of necrosis [1]. The conventional therapy includes surgical intervention, chemotherapy, and radiotherapy for low-grade gliomas [2], its median survival time is nearly 5 years starting from the diagnosis [3, 4]. High-grade gliomas are the frequently primary malignant glial tumors without the effective therapeutic strategy. In clinical practice, therapy strategy generally consists of surgical resection followed by chemotherapy and/or radiotherapy for high-grade gliomas. Although the improvement of traditional therapeutic modalities has only a little impact on the prognoses, the unfavorable prognosis remains to not be avoided in patients with high-grade gliomas [5, 6], and the median survival time is 7 months to 12 months for high-grade gliomas [7, 8].

In the past decades, molecular targeted therapies are beneficial to improve the prognosis of gliomas, such as bevacizumab, a monoclonal antibody against vascular endothelial growth factor (VEGF) [9], nimotuzumab, a monoclonal antibody to epidermal growth factor receptor (EGFR) [10] and several patients of gliomas with silencing of O6-methylguanine DNA methyltransferase (MGMT) could aggrieve more benefits from temozolomide (TMZ) [11]. Nevertheless, there could not be the molecular aberration in several gliomas patients. Therefore, it is necessary to explore new therapeutic approaches for the improvement of clinical prognosis in patients with gliomas, such as immunotherapy of anti-glioma-associated antigen (GAA) epitopes combining with poly ICLC have be investigated in the phase I study [12].

Programmed cell death-1 (PD-1)/Program death-ligand 1(PD-L1) pathway is a classic immune checkpoint of promising immunotherapeutic strategies. PD-L1 could help tumor cells immune evasion in combination with immunomodulatory properties [13]. Blockage of PD-L1 expression on tumor cells might activate tumor-specific T cell to kill tumor cells by mediating tumor necrosis factor alpha (TNF-α) and interferon gamma (IFN-γ) [14, 15]. PD-L1 expression was existed in several malignancies, such as cancers of the breast, pancreas, lung, renal and stomach [16, 17], and also some studies demonstrated that PD-L1 expression on tumor cells was correlated with unfavorable prognosis including non-small lung cancers, colorectal and breast cancers [18-20]. Meanwhile, several literatures showed that PD-L1 expression was upregulated in gliomas and a correlation was indicated between tumor grade and PD-L1 expression [21, 22]. However, there are rare studies on the prognostic value of PD-L1 in gliomas. In this study, we investigated the expression status of PD-L1 protein and the relationship between PD-L1 expression on tumor cells and prognosis of patients with gliomas by tissue-microarray-based immunohistochemistry.

## RESULTS

### Patients' features

The clinicopathological features of 229 patients were demonstrated in Table 1. This cohort included 125 (54.6%) males and 104 (45.4%) females, and median age was 50 years. Ninety-three patients (40.6%) were at early stage (grade I and II), and the other 136 patients (59.4%) were at late stage (grade III and IV). Average follow-up time was 65.8 months ranged from 1.0 to 142.0months (median, 36.0 months). Previous studies showed that the median survival time was 7 months to 12 months for patients with high-grade gliomas. Therefore, we divided the 229 patients into two groups, including the group of overall survival less than 12 months (short-time survival or follow up) and the group of that more than or equal to 12 months (long-time survival or follow up).

**Table 1:** Relationship between the PD-L1 and clinicopathological features in the 229 patients

	All cases	PD-L1 expression(<12 months)			All cases	PD-L1 expression(≥12 months)		
		negative	positive	P value		negative	positive	P value
Gender				0.861				0.355
Female	30	18(60.0%)	12(40.0%)		74	36(48.6%)	38(51.4%)	
Male	38	22(57.9%)	16(42.1%)		87	36(41.4%)	51(58.6%)	
Age at diagnosis (years)				0.881				0.454
> 50	42	25(59.5%)	17(40.5%)		130	60(46.2%)	70(53.8%)	
≥ 50	26	15(57.7%)	11(42.3%)		31	12(38.7%)	19(61.3%)	
Tumor site				0.831				0.260
Supratentorial	59	35(59.3%)	24(40.7%)		140	65(46.4%)	75(53.6%)	
Infratentorial	9	5(55.6%)	4(44.4%)		21	7(33.3%)	14(66.7%)	
Tumor recurrence				0.730				0.964
0	47	27(57.4%)	20(42.6%)		92	41(44.6%)	51(55.4%)	
1	21	13(61.9%)	8(38.1%)		69	31(44.9%)	38(55.1%)	
Pathological grade				0.798				0.327
I	0	0	0		10	4(40.0%)	6(60.0%)	
II	18	10(55.6%)	8(44.4%)		65	33(50.8%)	32(49.2%)	
III	20	11(55.0%)	9(45.0%)		54	25(46.3%)	29(53.7%)	
IV	30	19(63.3%)	11(36.7%)		32	10(31.2%)	22(68.8%)	
Chemotherapy				0.107				0.832
0	22	16(72.7%)	6(27.3%)		82	36(43.9%)	46(56.1%)	
1	26	24(52.2%)	22(47.8%)		79	36(45.6%)	43(54.4%)	
Radiotherapy				0.625				0.340
0	15	8(53.3%)	7(46.7%)		25	9(36.0%)	16(64.0%)	
1	53	32(60.4%)	21(39.6%)		136	63(46.3%)	73(53.7%)	

### The relationship between PD-L1 expression and patients’ clinicopathological features

In the study, the correlation of PD-L1 expression with the clinicopathological features was demonstrated in Table 1. The rate of PD-L1 expression was 51.1% (117/229) in all the patients with gliomas. For the two groups including short-time survival or follow up and long-time survival or follow up, there was no significant association between PD-L1 expression and all the clinicopathological features, such as age, gender, tumor site, pathological grade, tumor recurrence, presence or absence of chemotherapy and radiotherapy (P > 0.05, Table 1). However, PD-L1 expression rate was 49.2%, 53.7% and 68.8% for grade II, III and IV, respectively. Although no significant discrepancies was displayed, there was a certain degree of differences between PD-L1 expression and pathological grade (49.2% vs. 53.7% vs. 68.8%, P = 0.327).

**Figure 1:**
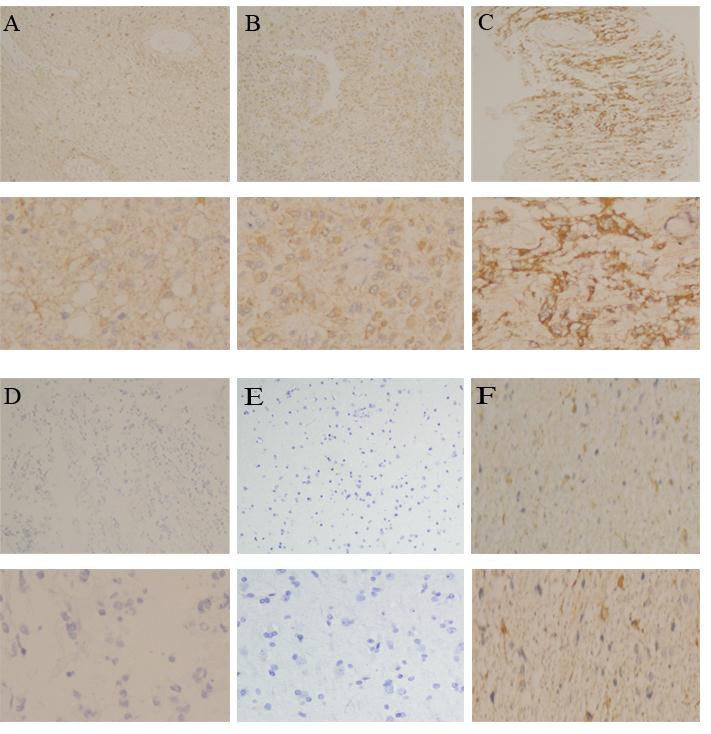
The expression pattern of PD-L1 in gliomas **A**. Weak expression of PD-L1 was shown in a glioma case (100×), **B**. Moderate expression of PD-L1 was shown in a glioma case (100×), **C**. Strong expression of PD-L1 was shown in a glioma case (100×), **D**. Negative expression of PD-L1 was shown in a glioma case (100 ×), **E**. Negative expression of PD-L1 was shown in normal brain tissue (100 ×), and **F**. 5% positive expression of PD-L1 was shown in a glioma case (100 ×) The lower panels indicated the higher magnification (200×) from the upper panels.

### The relationship between PD-L1 expression and prognosis of patients with gliomas

**Figure 2:**
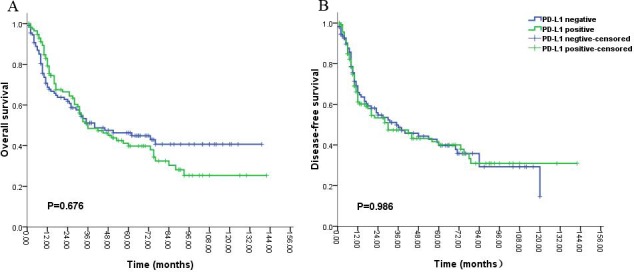
Kaplan-Meier survival analysis of PD-L1 expression and the prognosis including overall survival (A) and disease-free survival (B) for all the patients with gliomas

**Figure 3:**
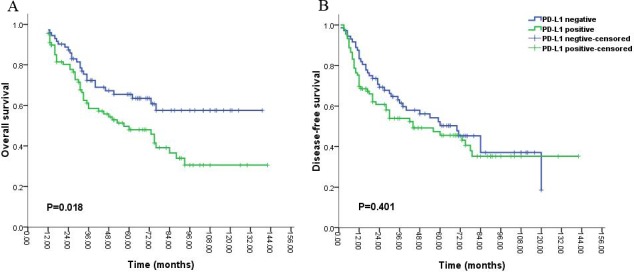
Kaplan-Meier survival analysis of PD-L1 expression and the prognosis including overall survival (A) and disease-free survival (B) for all the patients with gliomas during long-time survival or follow up

**Figure 4:**
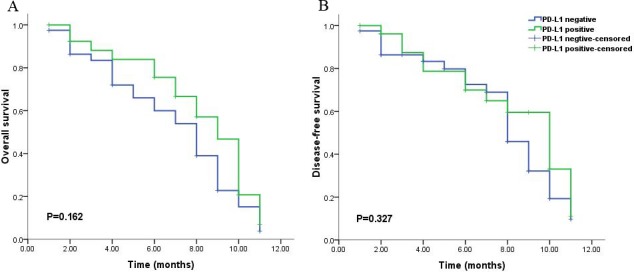
Kaplan-Meier survival analysis of PD-L1 expression and the prognosis including overall survival (A) and disease-free survival (B) for all the patients with gliomas during short-time survival or follow up

Univariate analysis showed that PD-L1 expression was not significantly correlated with OS (median OS; 36 months vs. 40 months, P = 0.676, Figure 2A) and DFS (median DFS; 75 months vs. 70 months, P = 0.988, Figure 2B) in 229 patients. According to the median survival time of 12 months for patients with glioblastoma (grade IV) in published previous studies, we divided the 229 patients into two groups including the group of short-time survival or follow up (OS < 12 months) and that of long-time survival or follow up (OS ≥ 12 months). For the group of patients with short-time survival or follow up, clinicopathological prognostic factors, (i.e. gender, tumor site, age, pathological grade, chemotherapy after surgery, radiotherapy after surgery and PD-L1 expression) were not inversely associated with OS and DFS (P > 0.05). A strong tendency towards the statistical significance was demonstrated between PD-L1 expression and better prognosis in the patients with the short-time survival or follow up (P = 0.162, Figure 4A). While the partial prognostic factors was closely linked with OS and DFS (P < 0.05, Tables 2 and 3) including tumor site, age, pathological grade, chemotherapy after surgery for the patients with long-time survival or follow up. Meanwhile, PD-L1 expression had a significant impact on the poor OS (P = 0.020, Table 2 and Figure 3A), but was not associated with DFS (P = 0.582, Table 3 and Figure 3B) in patients with long-time survival or follow up. PD-L1 expression and other clinicopathological features which were significant in univariate analysis were re-analyzed in multivariate analysis, including tumor site, age, pathological grade, chemotherapy after surgery and PD-L1 expression (Tables 2 and 3). Survival assessment revealed that PD-L1 expression was not correlated with poor OS (Cox regression model, hazard ratio: 1.536, 95% CI: 0.949-2.488, P = 0.081, Table 2), suggesting a strong tendency towards statistical significance between PD-L1 expression and adverse OS. However, age and pathological grade was served as independent prognostic predictors for OS and DFS (P < 0.05, Table 2 and 3).

**Table 2:** Univariate and multivariate analyses of different prognostic variables for overall survival in 161 patients with OS ≥12 months

Variable	Univariate analysis*			Multivariate analysis*	
	All cases	Hazard ratio (95% CI)	*P* value	Hazard ratio (95% CI)	*P* value
Gender			0.708		
Female	74	Reference			
Male	87	0.916 (0.580-1.448)			
Age at surgery (years)			**0.000**	3.327 (2.004-5.526)	**0.000**
< 50	130	Reference			
≥50	31	3.804 (2.324-6.225)			
Tumor site			**0.008**	1.950 (0.571-6.665)	0.287
Supratentorial	140	Reference			
Infratentorial	21	4.851 (1.524-15.447)			
Pathological grade			**0.000**	2.967 (1.720-5.149)	**0.000**
I-II	75	Reference			
III -IV	86	3.768 (2.254-6.301)			
Chemotherapy			**0.001**	1.387 (0.843-2.283)	0.198
0	82	Reference			
1	79	2.173 (1.351-3.494)			
Radiotherapy			0.205		
1	136	1.540 (0.790-3.003)			
PD-L1 expression			**0.020**	1.536 (0.949-2.488)	**0.081**
0	72	Reference			
1	89	1.760 (1.092-2.838)			

* Cox regression model; CI, confidence interval.

**Table 3:** Univariate and multivariate analyses of different prognostic variables for disease-free survival in 161 patients with OS ≥12 months

Variable	Univariate analysis*			Multivariate analysis*	
	All cases	Hazard ratio (95% CI)	*P* value	Hazard ratio (95% CI)	*P* value
Gender			0.054		
Female	74	Reference			
Male	87	0.620 (0.381-1.007)			
Age at surgery (years)			**0.001**	2.438 (1.401-4.244)	**0.002**
< 50	130	Reference			
≥50	31	2.589 (1.513-4.428)			
Tumor site			**0.048**	0.850 (0.329-2.197)	0.737
Supratentorial	140	Reference			
Infratentorial	21	2.341 (1.008-5.440)			
Pathological grade			**0.001**	1.779 (1.036-3.058)	**0.037**
I-II	75	Reference			
III -IV	86	2.408 (1.454-3.989)			
Chemotherapy			**0.000**	3.008 (1.685-5.371)	**0.000**
0	82	Reference			
1	79	3.635 (2.150-6.146)			
Radiotherapy			0.851		
0	25	Reference			
1	136	0.944 (0.515-1.729)			
PD-L1 expression			0.582		
0	72	Reference			
1	89	1.143 (0.711-1.837)			

* Cox regression model; CI, confidence interval.

### Stratified analysis

**Figure 5:**
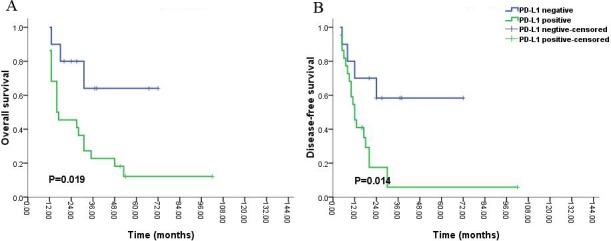
Kaplan-Meier survival analysis of PD-L1 expression and the prognosis including overall survival (A) and disease-free survival (B) for all the patients with glioblastoma (grade IV) during long-time survival or follow up

Stratified analysis revealed that PD-L1 expression was significantly associated with adverse OS (P = 0.019, Figure 5A) and DFS (P = 0.014, Figure 5B) for patients with grade IV gliomas during the long-time survival or follow up. However, no inversely correlations were found between PD-L1 expression and prognosis of patients with grade I, II, III during the long-time survival or follow up (P > 0.05).

## DISCUSSION

There were no critical advances for the treatment of high-grade gliomas in the past decades. Surgery combining with chemotherapy and radiotherapy remained to be the standard therapeutic strategies. Patients with high-grade gliomas, especially for whom was not sensitive to chemotherapy and radiotherapy, still had the poor prognosis [23]. Whereas the utility of molecular targeted agents in combination with chemoradiotherapy could improve the survival of patients with gliomas, and a degree of side effects were not avoided [24]. Therefore, further therapeutic approaches that assessed potential combinations of existing methods of treatments are urgently needed. In the recent years, Regulatory T cells and tumor-associated PD-L1 expression played an important role in the treatment of melanoma [25]. The promising immunotherapy of gliomas could depend on the inhibition of immune checkpoint.

In this study, we firstly reported that the intensity of PD-L1 expression in all the patients was weak or moderate positive, and PD-L1 expression was predominantly displayed on the cytoplasm and rarely presented in cellular membranes. These conclusions were partially consistent with a previous study [21]. It found that patients with high-grade gliomas had strong staining compared with those of low-grade gliomas for PD-L1 expression. These contradictory conclusions might be due to two reasons. One explanation was that different commercial anti-PD-L1 antibody had an impact on the conclusion, but our TMAs were not used as diagnosis standard of gliomas and could under-represent heterogeneity. The additional reason was that the majority of enrolled patients with high-grade gliomas could rarely have gene loss or mutation including PTEN, which contributed to upregulation of PD-L1 expression [22] as well as different response to immune activity in the tumor microenvironment could lead to the non-uniform PD-L1 expression of tumor cells [26]. Meanwhile, we also found that PD-L1 expression rate of tumor cells was 51.1% in all patients with gliomas. PD-L1 expression rate was 49.2%, 53.7% and 68.8% for grade II, III and IV, respectively. Although no significant discrepancies was displayed, there was a certain degree of differences between PD-L1 expression and pathological grade (49.2% vs. 53.7% vs. 68.8%, P = 0.327). This finding suggested that patients with high-grade gliomas might have the high PD-L1expression resulted in immunoresistance to the immunotherapy. There was a significant association between PD-L1 expression and disease progression in lung cancers and gastric cancers [27, 28]. Although the prognostic impact of PD-L1 expression in patients with carcinomas remained controversial. However, our finding still had some clinical significance. We speculated that different commercial anti-PD-L1 antibody and different immunohistochemical method of PD-L1 staining could have the effect on this result, and the TMAs of gliomas remained not to represent the grossly tumor tissues. In addition, cutoff values differentiating high or low level of PD-L1 expression assessed by IHC evaluation were varied. Therefore, these limitations might have a degree of impact on our findings.

Some studies demonstrated that PD-L1 expression on tumor cells was significantly associated with adverse prognosis in various malignancies [29-31], and other studies revealed that PD-L1 expression on tumor cells was significantly associated with better prognosis in several malignancies [32-34]. Interestingly, we found that no significant association between PD-L1 expression and overall survival was observed in all the patients with gliomas, which was in agreement with prior clinical trials. These studies revealed that melanoma patients with negative expression of PD-L1 in the tumors also displayed objective responses to nivolumab in a Phase I clinical study[35] and no relationships were found between positive expression of PD-L1 improved responses to nivolumab and ipilimumab in another Phase I clinical trials [36]. This finding suggested that PD-L1/PD-1 pathway contributed to only a little role on the effect of prognosis in various interplay anti-tumor immune mechanisms, including the mediation of Transforming Growth Factor β (TGF-β), Macrophage Chemoattractive Protein 1 (MCP-1), Cytotoxic T-Lymphocyte Antigen-4 (CTLA-4) and Fas Receptor (FasR)/Ligand (FasL) pathway [37]. However, we verified that a statistical tendency towards the relationship between PD-L1 expression and better prognosis in the patients with the short-time survival or follow up (OS < 12 months). We speculated that the possible immune mechanism was tumor infiltration by T cells did not expression PD-1co-receptor [38] and tumor cells could be killed by CD8+ T cells or NK cells mediated by interferon-γ and interleukin-12 [39]. Meanwhile, a significant correlation of PD-L1 expression with poor prognosis was demonstrated in the patients with long-time survival or follow up (OS ≥ 12 months). To the best of our knowledge, the expression of PD-L1 was displayed in various malignancies, and tumor cells might increase the expression of PD-L1, interaction with PD-1 produced by activated T lymphocytes was able to make tumor cells escape from immune destruction [40]. This finding was in agreement with our study that a significant association between PD-L1 expression and worse prognosis in the patients with long-time survival or follow up. Notably, our data demonstrated that the complicated relationship of PD-L1 expression with the survival of patient, it was possible that PD-L1 was identified as an inducible indicator, and could not simply be considered as other molecular makers in tumors, various methods were used to detect the expression of PD-L1 by immunohistochemistry with numerous antibodies, and the precise cutoff values of PD-L1 expression by immunohistochemistry were still not be defined for varied tumors in the published article, thereby also indicated that the unidentified cutoff value of PD-L1 expression had an effect on the results [41]. The conflicting results were not contradictory, some mechanisms could be controlled concordantly, and their related interactions might vary with different tumor type.

As far as we known, the inhibition of PD-1/PD-L1was recommended as a therapeutic agent, specific monoclonal antibodies of PD-1 and PD-L1 also were successfully applied and more development was gained in phase I clinical trials [42, 43]. Nonetheless, no therapeutic response was observed in the patients with gliomas. In our study, expression frequency of PD-L1 was 51.1% and a significant association was confirmed between PD-L1 expression and worse prognosis in the patients with gliomas during long-time survival or follow up, and stratified analysis revealed that PD-L1 expression was significantly associated with adverse OS and DFS for patients with gliomas of grade IV in these patients, suggesting that the immunotherapy of PD-1 and PD-L1 could be effective in the patients with gliomas, especially in the patients with grade IV to further improve the poor survival time.

Our study still had several limitations. Firstly, this study was a respective analysis. Secondly, PD-L1 expression was assessed by immunohistochemistry in TMAs and the information of tumor tissue could not be fully reflected in TMAs.

In summary, we have found that expression frequency of PD-L1 was displayed in 51.1% patients with gliomas and was correlated with worse prognosis in the patients with long-time survial or follow up. However, a statistical tendency was found between PD-L1 expression and better prognosis in the patients with short-time survival or follow up. Although a study found patients of grade IV gliomas with PD-L1 expression had a poor prognosis, the number of 17 samples was too rare [44]. Therefore, further studies were needed to investigate the complex role of PD-L1 expression and the treatment effect of inhibition of PD-1 and PD-L1 in large scale clinical trials for patients with gliomas.

## MATERIALS AND METHODS

### Ethical approval

This study was supported by Sun Yat-sen University Cancer Center ethics committee. No informed consent (written or verbal) was achieved for the retrospective analysis. Part of the enrolled patients was dead. Therefore, it was not deemed to be necessary by the ethics committee, and all samples were anonymous.

### Patients

In the study, we collected information for clinicopathological features and survival time in patients with gliomas, 229 samples were achieved in Sun Yat-Sen University Cancer Center from January 2000 to August 2008. The screening of specimens was suitable for the following conditions: gliomas was diagnosed by 2008 World Health Organization classification of central nervous system tumors; The complete clinicopathological data included age, gender, tumor site, histological subtype, pathological grade; neither previous malignancies nor an secondary tumor; without neoadjuvant chemotherapy (IC), radiotherapy (RT) and chemoradiotherapy (CRT) before surgical resection, follow-up regularly. The Institute Research Medical Ethics Committee of Sun Yat-Sen University granted approval for this study.

### Construction of the tissue microarray (TMA)

Tissue microarrays were constructed in accordance with a previously described method [45]. In brief, the paraffin-embedded tissue blocks and the corresponding histological H&E-stained slides were overlaid for tissue TMA sampling. Duplicate of 0.8 mm diameter cylinders were punched from representative tumor areas of individual donor tissue block, and re-embedded into a recipient paraffin block at a defined position, using a tissue arraying instrument (Beecher Instruments, Silver Spring, MD, USA).

### Immunohistochemistry (IHC)

Formalin-fixed, paraffin-embedded gliomas samples were cut into 4-μm thick sequential sections and processed for IHC according to the previously described protocol [46]. The TMA slides were deparaffinized in xylene, rehydrated through graded alcohol, immersed in 3% hydrogen peroxide for 10 min to block endogenous peroxidase activity and antigen retrieved by pressure cooking for 3 min in citrate buffer (pH = 6). For blocking nonspecific binding, the slides were preincubated with 10% normal goat serum at room temperature for 20 min. Subsequently, the slides were incubated with rabbit polyclonal antibody anti-PD-L1 (1:100 dilution), overnight at 4°C in a moist chamber. The slides were sequentially incubated with a secondary antibody (Envision, Dako, Denmark) for 30 min in the incubator at 37°C, and stained with DAB (3,3-diaminobenzidine). Finally, the sections were counterstained with Mayer's hematoxylin, dehydrated and mounted. A negative control was obtained by replacing the primary antibody with a normal rabbit IgG.

### IHC evaluation

The expression status of PD-L1 was evaluated by microscopic observation of stained TMA slides. The staining of cytoplasm or membranes was deemed to be positive for PD-L1 expression in over 5% tumor cells [34]. PD-L1 expression was evaluated independently by X-K Zhang and S-Y Xi with more than 90% concordance.

### Statistical analysis

Statistical analysis was performed with SPSS software, version 16.0 (SPSS, Chicago, USA). The correlation between PD-L1 expression and clinicopathological features was assessed by chi-square test. Kaplan–Meier method was used to evaluate the relationship between survival time and clinicopathological parameters. Survival time included overall survival (OS: the date of surgery to the date of death from any cause, or to the last follow-up date if the patient is alive) and disease-free survival (DFS: the length of time from the date of surgery on the primary tumor to local, regional, or distant recurrence or death from any cause). The Cox proportional hazards regression model was used to define independent prognostic biomarkers that inversely impacted OS and DFS. A two-tailed P-value less than 0.05 was served as statistically significant.
